# Dendritic cells, macrophages, NK and CD8^+^ T lymphocytes play pivotal roles in controlling HSV-1 in the trigeminal ganglia by producing IL1-beta, iNOS and granzyme B

**DOI:** 10.1186/s12985-017-0692-x

**Published:** 2017-02-21

**Authors:** Natália Lucinda, Maria Marta Figueiredo, Natália Lima Pessoa, Beatriz Senra Álvares da Silva Santos, Graciela Kunrath Lima, Arthur Molinari Freitas, Alexandre Magalhães Vieira Machado, Erna Geessien Kroon, Lis Ribeiro do Valle Antonelli, Marco Antônio Campos

**Affiliations:** 1 Imunologia de Doenças Virais, Centro de Pesquisas René Rachou, Fundação Oswaldo Cruz, Fiocruz, Avenida Augusto de Lima 1715, Belo Horizonte, 30190-002 MG Brazil; 2 Escola de Veterinária, Universidade Federal de Minas Gerais, Avenida Antônio Carlos 6627, Belo Horizonte, 31270-901 MG Brazil; 3 Laboratório de Vírus, Departamento de Microbiologia, Universidade Federal de Minas Gerais, Avenida Antônio Carlos 6627, Belo Horizonte, 31270-901 MG Brazil; 4 Biologia e Imunologia Parasitária, Centro de Pesquisas René Rachou, Fundação Oswaldo Cruz, Fiocruz, Avenida Augusto de Lima 1715, Belo Horizonte, 30190-002 MG Brazil

**Keywords:** Herpes simplex virus 1, Innate immunity, Dendritic cells, Macrophages, CD8^+^ T lymphocytes, TLRs, Murine model, Neuropathogenesis, Encephalitis

## Abstract

**Background:**

Herpes simplex virus type 1 (HSV-1) cause not only mild symptoms but also blindness and encephalitis. It was previously shown that the immune response against HSV-1 occurs mainly in the trigeminal ganglia (TG) and that Toll-like receptors 2 and 9 (TLR2/9) are important in mediating this response. It was also demonstrated that iNOS (nitric oxide synthase) and interleukin 1 beta (IL-1β) play an essential role in the defense against HSV-1 infection. Importantly, the present work aimed to identify the primary cells responsible for iNOS and IL-1β production and search for other important molecules and cells that might or might not depend on TLR2/9 receptors to mediate the immune response against HSV-1.

**Methods:**

C57BL/6 (wild type, WT) and TLR2/9^−/−^ mice were infected by the intranasal route with HSV-1 (1 × 10^6^ p.f.u.). Cells were obtained from the TG and spleen tissues and the profile of immune cells was determined by flow cytometry in infected and mock infected WT and knockout mice. The percentage of cells producing iNOS, IL-1β, granzyme B and perforin was also determined by flow cytometry. Chemokine monocyte chemoattractant protein-1 (MCP1) was measured by Cytometric Bead Array (CBA) in the TG, spleen and lung. Expression of type I interferons (IFNs), interleukins (IL) 5 and 10, IL-1β and granzyme B were quantified by real time PCR.

**Results:**

The results indicate that dendritic cells (DCs) and monocytes/macrophages (Mo/Mϕ) were the main sources of IL-1β and iNOS, respectively, which, together with type I IFNs, were essential for the immune response against HSV-1. Additionally, we showed that granzyme B produced by CD8^+^ T and NK lymphocytes and MCP-1 were also important for this immune response. Moreover, our data indicate that the robust production of MCP-1 and granzyme B is either TLR-independent or down regulated by TLRs and occurs in the TG of TLR2/9^−/−^ infected mice.

**Conclusion:**

Taken together, our data provide strong evidence that the responses mediated by DCs, Mo/Mϕ, NK and CD8^+^ T lymphocytes through IL-1β, iNOS and granzyme B production, respectively, together with the production of type I IFN early in the infection, are crucial to host defense against HSV-1.

**Electronic supplementary material:**

The online version of this article (doi:10.1186/s12985-017-0692-x) contains supplementary material, which is available to authorized users.

## Background

Herpes simplex virus type 1 (HSV-1) usually causes mild clinical symptoms such as herpes labialis, but in neonates and immunocompromised individuals, HSV-1 could lead to severe ophthalmic and neurologic lesions, culminating in blindness, encephalitis or even death [1]. HSV-1 establishes a lifelong latent infection in neuronal cells, predominantly in trigeminal ganglia (TG) [2]. The innate immune response is generated mainly through pattern recognition receptors (PRRs), such as Toll-like receptors (TLRs), which are capable of recognizing pathogen-associated molecular patterns (PAMPs) [3]. Studies have shown that deficiency in some TLRs and their transducers, such as MyD88 (myeloid differentiation primary response 88) and TRIF (TIR-domain-containing adapter-inducing interferon-β), can result in encephalitis and host death [4–9]. Infectious agent recognition activates TLR-signaling pathways, which culminate in pro-inflammatory cytokine production [3, 10, 11]. Among these cytokines, type I interferons (IFNs) (mainly IFN-α and IFN-β) play an important role in the innate response to HSV-1, since they stimulate the expression of many interferon-stimulated genes. This stimulation results in the production of cytokines, such as interleukin 1 beta (IL-1β), tumor necrosis factor alpha (TNF-α), IFN-γ and some chemokines, that lead to the recruitment of innate immune cells to the local site of infection, ending ultimately in activation of the adaptive response and infection control [12–16]. The main cells responsible for this coordinated immune response against HSV-1 are natural killer (NK), dendritic (DC) and T cells, mainly CD8^+^ lymphocytes [10, 11].

Thus, to study the immune response triggered by this herpesvirus, we used the intranasal model of infection. This route of infection was an alternative to the labial infection since this method caused lesions in the oral epithelium that resulted, ultimately, in cannibalism among the animals. As the TG innervates extensive areas of the face (through branches of the ophthalmic, maxillary and mandibular nerves), including the oral and nasal region [17–19], this model of infection maintain the route of HSV-1 infection and does not cause tissue lesions.

Previous studies from our group showed that the TG, but not the brain, seem to be the local site where an efficient immune response against HSV-1 occurs [6, 8, 9]. We used the HSV-1 strain EK, which was isolated from a human case of recurrent oral herpes with blisters. C57BL/6 intranasally infected with 10^6^ pfu of this strain show mild signs of infection (fur loss and snout edema) by the 4^th^ and 5^th^ day after infection. C57BL/6 mice have a mortality rate of only 10% while TLR2/9^−/−^ mice have a 100% mortality rate between the 5^th^ and 8^th^ day after infection with signs of encephalitis (prostration, ruffled fur, hunched posture, and posterior paw paralysis) [6, 8].

Analysis of the kinetics of viral replication as well as of some cytokines expression in the TG of C57BL/6 animals revealed that on the 5^th^ day post infection (5 dpi) there are peaks of virus multiplication and also of some pivotal cytokines expression for an efficient immune response [8]. These peaks are subsequently accompanied by a decrease in viral replication as well as in the levels of cytokines. Additionally, no viral particles were detected in the brains of these animals, showing that they can control the infection in the TG, not developing encephalitis [8]. For this reason in our model of intranasal infection we have chosen the 5^th^ day to study the immune response, since, it seems to be the “key” day to control virus in the TG of C57BL/6 animals in this model. Thus, immune response analyzes in TLR2/9^−/−^ animals were also performed on this day as they beginning to die after the 5^th^ day, which suggests that this day is important for the infection outcome. Moreover, it was demonstrated that the adaptor molecule MyD88 or the TLR2 and TLR9 receptors together are crucial to host survival, since mice deficient in these molecules succumb to HSV-1 infection with encephalitis, whereas wild-type (WT) C57BL/6 mice can control the infection and survive [8, 9]. We have also demonstrated the important role of macrophages (Mϕ) as iNOS producers in host defense against HSV-1 [6].

Here, the present work aimed to further study the immune response against HSV-1 by identifying the organs and cells responsible for producing iNOS, IL-1β and granzyme B in WT mice, together with the early production of type I IFN, and comparing them to the response in TLR2/9^−/−^ mice to identify which molecules are dependent on TLR2 and TLR9 receptors together and looking for other important organs and molecules in the HSV-1 immune response.

## Methods

### Vero cells

For virus replication and titration, Vero cells (ATCC) were maintained in Dulbecco’s Modified Eagle’s Medium (DMEM) (Sigma) supplemented with 5% heat-inactivated fetal bovine serum (FBS) (Cultilab, Brazil) and antibiotics at 5% CO_2_ and 37 °C.

### Virus replication and purification

Cell culture flasks containing cells at 80% confluence were infected with HSV-1, strain EK [20], which was isolated from a human case of recurrent oral herpes with blisters, at a multiplicity of infection (m.o.i.) of 0.1. After virus adsorption for 1 h in DMEM, the cells were maintained for 2 days in DMEM supplemented with 2% FBS. Purification of infectious virus particles was performed by collecting the supernatant of infected Vero cells. The supernatants were precipitated by dripping a saturated ammonium sulfate solution to yield an ammonium sulfate concentration of 60% in the final volume of the mixture. This step occurred on ice under agitation. The mixture was then centrifuged at 10,415 × g at 4 °C for 30 min. The precipitate was dissolved in 5 mL of 10 mM Tris-HCl, pH 8.0, and centrifuged in a sucrose gradient (36%) in an ultracentrifuge at 36,320 × g at 4 °C for 2 h. The supernatants were carefully removed and the pellet was dissolved in Tris-HCl 10 mM, pH 8.0.

### Animals

**Fig. 1:**
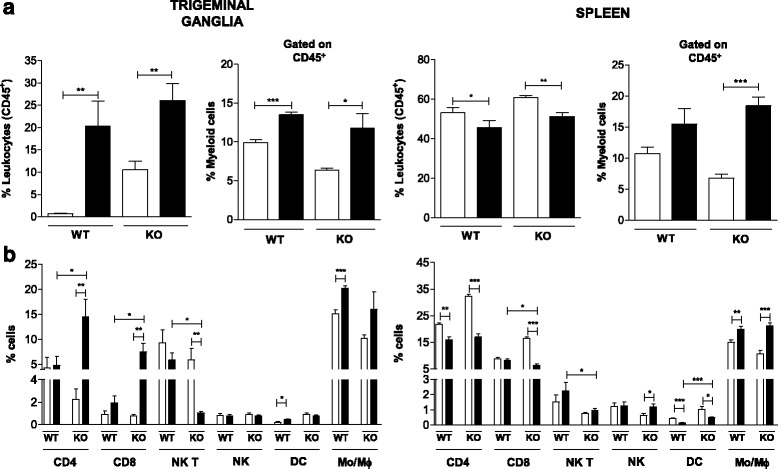
DCs and monocyte/macrophage are higher in C57BL/6 infected than in mock-infected mice, differently of TLR2/9^−/−^. The profile of immune cells present in the *TG* and *spleen* of C57BL/6 (WT) and TLR2/9^−/−^ (KO) mice was assessed by cytometry analysis. Groups of six mice were infected with 10^6^ p.f.u. of HSV-1 via the intranasal route and, on the 5^th^ day post infection, mice were euthanized, and *TG* and *spleen* cell suspensions were prepared. **a** Frequencies of live leukocytes (CD45^+^) were analyzed within the population of *TG* and *spleen* cells and frequencies of myeloid cells (CD45^+^CD3^−^CD11b^+^) were analyzed within the population of live leukocytes in the TG and spleen; **b** CD4 (CD45^+^CD3^+^CD4^+^), CD8 (CD45^+^CD3^+^CD8^+^), NK T (CD45^+^CD3^+^NK1.1^+^), NK (CD45^+^NK1.1^+^), dendritic (DC, CD45^+^MHC class II^high+^CD11c^+^) and monocyte/macrophage (Mo/Mϕ, CD45^+^F4/80^+^) cells were analyzed within the population of live leukocytes in the *TG* and *spleen*. A minimum of 100,000 events was acquired for analysis. *Bars* show the means ± SEM. * *p* < 0.05; ** *p* < 0.01 and *** *p* < 0.001. *White bars*: mock-infected mice; *black bars*: infected mice

**Fig. 2:**
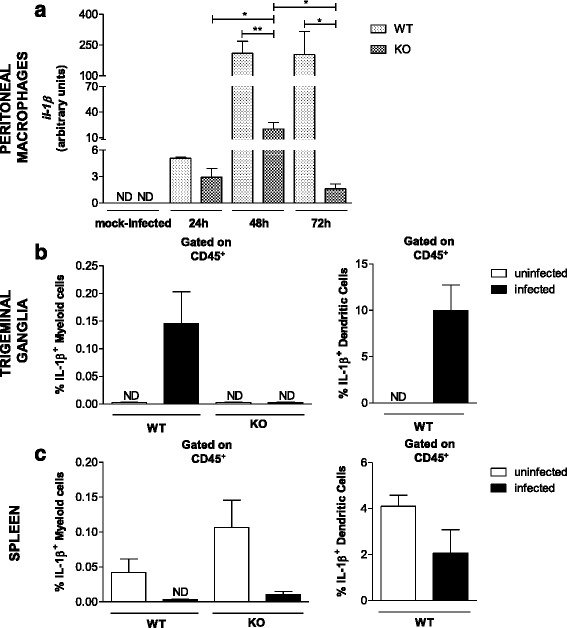
DCs are the major producers of IL-1β in the TG of C57BL/6 mice after infection. **a** Peritoneal macrophages derived from C57BL/6 (WT) and TLR2/9^−/−^ (KO) mice were infected with HSV-1 (m.o.i. of 1, 5 wells/group), and the levels of IL-1β mRNA were determined in the supernatants at different times after infection (as indicated in the figure) by qPCR. The results use arbitrary units for the ratio of target gene mRNA to endogenous control HPRT mRNA. Data are representative of two independent experiments with similar results. For myeloid cell and DC frequency analyses, groups of six mice were infected with 10^6^ p.f.u. HSV-1 via the intranasal route and, on the 5^th^ day post infection, mice were euthanized, and TG and spleen cell suspensions prepared for flow cytometry evaluation. The frequencies of myeloid (CD45^+^CD3^−^CD11b^+^) and dendritic (CD45^+^MHC class II ^high+^CD11c^+^) cells producing IL-1β in the TG (**b**) and spleen (**c**) are shown. The frequency of each cell was calculated within the population of live leukocytes. A minimum of 100,000 events was acquired for analysis. *ND*: not detected. *Bars* show the means ± SEM. * *p* < 0.05 and ** *p* < 0.01

**Fig. 3:**
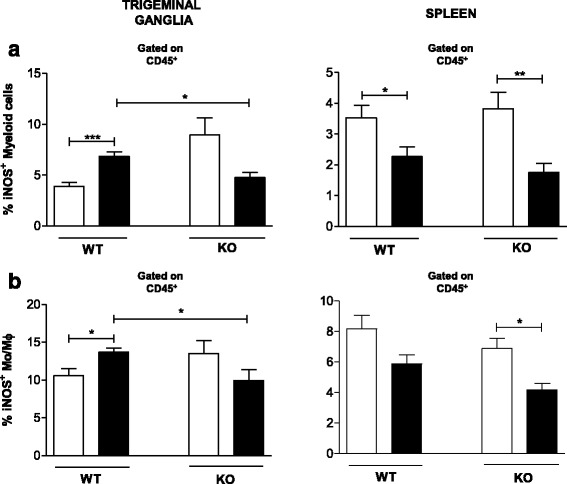
Monocytes/macrophages are the main iNOS producers in the TG of C57BL/6 mice during HSV-1 infection. Groups of six mice were infected with 10^6^ p.f.u. HSV-1 via the intranasal route and, on the 5^th^ day post infection, mice were euthanized, and TG and spleen cell suspensions prepared for cytometry analyses. **a** Frequencies of iNOS-producing myeloid cell populations (CD45^+^CD3^−^CD11b^+^) within the live leukocyte population. **b** Frequencies of iNOS-producing monocytes/macrophages (Mo/Mϕ) (CD45^+^F4/80^+^) within the population of live leukocytes in the TG and spleen. A minimum of 100,000 events was acquired for analysis. KO = TLR2/9^−/−^ mice. *White bars*: mock-infected mice; *black bars*: infected mice. *Bars* show the means ± SEM. * *p* < 0.05; ** *p* < 0.01 and *** *p* < 0.001

**Fig. 4:**
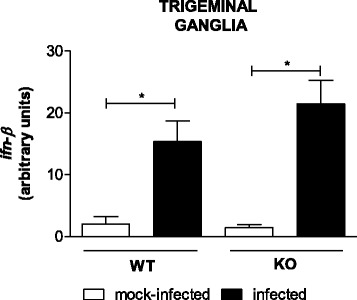
IFN-β expression occurs in the TG of both WT and TLR2/9^−/−^ animals after HSV-1 infection. Mice were infected with 10^6^ p.f.u. of HSV-1, euthanized on the 5^th^ day post infection, and the TGs were collected for mRNAs expression analysis by qPCR. The results use arbitrary units for the ratio of target gene mRNA to endogenous control HPRT mRNA. Data are representative of two independent experiments with similar results. KO = TLR2/9^−/−^ mice. *Bars* show the means ± SEM. * *p* < 0.05. *n* = 4–8 mice/group

The mouse colonies and all experimental procedures were performed according to the institutional animal care and use guidelines from CPqRR/FIOCRUZ. The project was approved by the Ethics Committee in Animal Experimentation (CEUA from CPqRR/FIOCRUZ LW6/11 and LW-20/15). TLR2 and TLR9 knockout mice (TLR2/9^−/−^) were obtained by crossing TLR2^−/−^ and TLR9^−/−^ mice (both generated at Osaka University, Japan) at the National Institutes of Health (NIH, USA) and by backcrossing them to the C57BL/6 background for eight generations. These mice were kind gifts of Shizuo Akira and Alan Sher, respectively. The C57BL/6 mice used as wild-type (WT) controls were obtained from the Centro de Pesquisas Renê Rachou, Oswaldo Cruz Foundation (CPqRR/FIOCRUZ) (Belo Horizonte, Minas Gerais, Brazil), where all of the mice were maintained in a pathogen-free barrier environment. Six- to 10-week-old male mice were anesthetized with ketamine and xylazine. The mice were intranasally infected with 1 × 10^6^ plaque-forming units (p.f.u.) of purified HSV-1 as described previously [21]. The control mice were administered phosphate-buffered saline (PBS). In the experiments in Figs. 1, 2b-c, 3, 4, 6, 7, 8 and 9, the mice were euthanized 5 days after infection, because a previously performed kinetic analysis of viral growth and the cytokine response showed that the peak of virus and cytokine levels occurred on the 5th day after infection when this multiplicity of infection was used [8].

### Intraperitoneal macrophages

Thioglycolate-elicited peritoneal macrophages were obtained from either C57BL/6 or TLR2/9^−/−^ mice by peritoneal washing. Adherent peritoneal macrophages were cultured in 6-well plates in an atmosphere with 5% CO_2_ at 37 °C in DMEM supplemented with 5% FBS and antibiotics. A group of wells were infected with HSV-1 at a m.o.i. of 1. A second group was used as a control and did not receive any stimulus. All wells were then activated with sub-optimal concentration of murine IFN-γ (20 U/mL). At different time points (24, 48 and 72 h post infection), the cells were harvested, and the supernatant was collected and homogenized in TRIzol Reagent (Invitrogen) for RNA isolation and subsequent reverse transcription (RT) reaction.

### RNA extraction and reverse transcription

Five days post infection, for the TG and spleen, and 3hs and 24hs post infection, for the lung tissues, were aseptically collected, homogenized in TRIzol (Thermo Fisher Scientific, USA) and stored at −70 °C until RNA extraction. RNA extraction was performed according to the procedures provided by the manufacturer of TRIzol reagent. Total RNA was treated with DNase (Promega, USA) and subjected to a RT reaction that used M-MLV enzyme (Promega, USA) according to the manufacturer's instructions.

### Quantitative PCR (qPCR)

qPCRs were performed to measure mRNA expression in the TG, lung, spleen tissues and peritoneal macrophages. The reactions were performed with SYBR Green PCR Master Mix (Applied Biosystems, USA) in a 7500 real-time PCR System (Applied Biosystems, USA). Amplification was performed with SYBR Green PCR Master Mix using the following conditions: 95 °C for 10 min, followed by 50 two-step cycles of 95 °C for 15 s and 60 °C for 1 min and a final dissociation stage. The qPCR was performed with oligonucleotide primer pairs specific for the coding region of hypoxanthine-guanine phosphoribosyltransferase (HPRT) (forward: 5’-GTT GGA TAC AGG CCA GAC TTT GTT G-3’, reverse: 5’-GAT TCA ACT TGC GCT CAT CTT AGG C-3’); granzyme B (GRZ-b) (forward: 5’-ACT TTC GAT CAA GGA TCA GCA-3’, reverse: 5’-GGC CCC CAA AGT GAC ATT TAT T-3’); IL-10 (forward: 5’-GGT TGC CAA GCC TTA TCG GA-3’, reverse: 5’-ACC TGC TCC ACT GCC TTG CT-3’); IL-5 (forward: 5’-ACA GTG GTG AAA GAG ACC TT-3’, reverse: 5’-TCC AAT GCA TAG CTG GTG ATT T-3’); chemokine (C-C motif) ligand 2 (CCL-2 or MCP-1) (forward: 5’-CTT CTG GGC CTG CTG TTC A-3’, reverse: 5’-CCA GCC TAC TCA TTG GGA TCA-3’) [22]; pro-interleukin 1 beta (pro-IL-1β) (forward: 5’-CGC AGC AGC ACA TCA ACA AGA GC-3’, reverse: 5’-TGT CCT CAT CCT GGA AGG TCC ACG-3’); interferon alpha 4 (IFN-α4) (forward: 5’- ACT CAT TCT GCA ATG ACC TCC A -3’, reverse: 5’- AGA GGA GGT TCC TGC ATC ACA -3’) and interferon beta (IFN-β) (forward: 5’-CTGGAGCAGCTGAATGGAAA-3’, reverse: 5’-TGTCTGCTGGTGGAGTTCAT-3’). A standard curve was prepared by serial dilution of the amplified DNA for each target gene. The standard curve method was used to analyze the data. The mRNA levels of the target genes were normalized to the expression of HPRT, the housekeeping gene. All reactions were done in duplicate.

### Cytokine bead array analysis

MCP-1 levels were measured in TG, lung, spleen and cervical lymph node tissues using Cytometric Bead Array kit (CBA, BD Biosciences Pharmingen, USA) according to the manufacturer’s instructions. The tissues from mock infected and infected C57BL/6 and TLR2/9^−/−^ mice were homogenized in PBS containing protease inhibitors (Complete mini [EDTA-free], Roche, Basel, Switzerland), and the supernatants were used for cytokine measurements.

### Isolation of TG and splenic leukocytes

Single-cell suspensions were harvested from the TG and spleens of six C57BL/6 and six TLR2/9^−/−^ mock-infected (PBS) and infected (1x10^6^ p.f.u.) mice, 5 days after infection. TG were incubated with 2 mg/mL collagenase (*Merck-Chemicals*) for 30 min in 5% CO_2_ at 37 °C. Single-cell suspensions were prepared by passing the TG and spleen through a 70-μm nylon cell strainer (BD Biosciences). Splenocytes were then treated with ACK lysis buffer (BD Biosciences) on ice for two minutes. Samples were suspended in complete Roswell Park Memorial Institute medium (RPMI) and plated for flow cytometry analysis.

### Flow cytometry analysis of cell populations and intracellular cytokine production

For flow cytometry analysis, BD GolgiPlug Protein Transport Inhibitor (BD Biosciences, San Jose, CA) was added, and cells were cultured for 10 h in 5% CO_2_ at 37 °C. After incubation, cells were washed with FACS buffer (PBS with 2% FBS) and stained with Acqua Live/Dead (Invitrogen, USA) for 10 min at 4 °C for dead cell exclusion. Cells were washed and stained for surface molecules for 30 min at 4 °C, fixed and permeabilized according to the manufacturer’s instructions (Cytofix/Cytoperm, BD Biosciences, USA). After washing, cells were incubated with antibodies against intracellular antigens for 30 min at 4 °C. Cells were then washed and suspended in 200 μL of FACS buffer for cytometry analysis. Data were collected using an LSR II (BD Immunocytometry Systems, USA) with Diva (BD Biosciences, USA). At least 100,000 gated events were acquired. Representative FACS density plots showing the gate strategy for the identification of IL-1β within CD11c^+^MHCII^high^ and iNOS within F4/80^+^ gated on live CD45^+^ leucocytes in the trigeminal ganglia and spleen from a single HSV1-infected wild type mouse were done on Additional file 1: Figure S1 and Additional file 2: Figure S2, respectively.

The antibody panels used to define the cell subpopulations included the following: anti-CD11c PE-Cy7 (clone N418), anti-F4/80 eFluor 450 (clone BM8), anti-CD45.2 Alexa Fluor 700 (clone 104), anti-NK1.1 APC-eFluor 780 (clone PK136), anti-IL-1β pro-form APC (clone NJTEN3), anti-granzyme B PE-Cy7 (clone 16G6) and anti-perforin FITC (clone eBioOMAK-D), all purchased from eBioscience, USA; anti-I-4/I-E biotin (clone 2G9), anti-CD8a PE (clone 53–6.7) and anti-CD3e PerCP (clone 145-2C11), purchased from BD Pharmingen, USA; streptavidin Qdot 605 and anti-CD4 Qdot 605 (clone RM4-5), purchased from Invitrogen, USA; anti-CD11b Brilliant Violet 570 (clone M1/70), purchased from BioLegend, USA; and anti-iNOS/NOS Type II FITC (clone 6/iNOS/NOS Type II), purchased from BD Transduction Laboratories, USA. Data were analyzed with FlowJo (Tree Star) software. The expression of myeloid markers was analyzed after gating on CD45^+^CD3^−^ live cells.

### Statistical analysis

The statistical analyses were performed using GraphPad Prism 5 software for Windows (GraphPad Software, Inc., La Jolla, CA, USA). The sample groups were assessed by non-parametric or parametric tests depending on Kolmogorov-Smirnov normality. The Mann–Whitney test was used for nonparametric data, and an unpaired *T* test was used for parametric data.

## Results

### More dendritic cells and monocytes/macrophages are present in the TG of infected WT mice than mock-infected WT mice, in contrast to TLR2/9^−/−^ mice

Cell population profiles in the TG and spleen of C57BL/6 and TLR2/9^−/−^ mice were analyzed without infection and on the 5^th^ day of HSV-1 infection using flow cytometry (Fig. 1). The percentage (%) of leukocytes (CD45^+^) and myeloid cells (CD45^+^CD3^−^CD11b^+^) increased in the TG of WT and TLR2/9^−/−^ mice after HSV-1 infection (Fig. 1a). In the spleen, the % of myeloid cells was also larger in the infected groups. However, the global leukocyte % decreased in the spleen in both groups after infection (Fig. 1a). Further analysis of the cell profile in the TG showed that the % of DCs and Mo/Mϕ was larger in infected WT mice than in mock-infected WT mice, while there were no significant alterations in the presence of these two cell types in infected and mock-infected TLR2/9^−/−^ mice (Fig. 1b). The % of CD4^+^ T and CD8^+^ T lymphocytes present did not differ significantly in infected and mock-infected WT mice, whereas these cell populations were larger in infected than mock-infected TLR2/9^−/−^ mice. In contrast, the % of NK T cells in the TG was smaller in infected than mock-infected TLR2/9^−/−^ mice, whereas the WT mice presented no significant difference in the % of this cell population in infected and mock-infected mice. The NK cells in the TG presented the same profile in WT and TLR2/9^−/−^ infected and mock-infected mice. In the spleen, the % of DCs and CD4^+^ T lymphocytes was smaller in infected than in mock-infected WT and TLR2/9^−/−^ mice. The % of CD8^+^ T lymphocytes was lower in the spleen of infected than mock-infected TLR2/9^−/−^ mice, but the profile of CD8^+^ T lymphocytes in the spleen of infected and mock-infected WT mice was similar. The number of NK T cells in the spleen did not change significantly with the infection of WT and TLR2/9^−/−^ animals, although the percentage of NK T cells in infected WT mice was larger than that in the TLR2/9^−/−^ mice. There were more NK cells in infected than mock-infected TLR2/9^−/−^ mice, while there was no difference between infected and mock-infected WT mice. Finally, in the spleen, there was a lower % of Mo/Mϕ in the mock-infected mice than in the infected WT and TLR2/9^−/−^ mice.

### Dendritic cells are the main producers of IL-1β in the TG and are crucial for HSV-1 defense

The kinetics of IL-1β mRNA transcription in peritoneal Mϕ from WT and TLR2/9^−/−^ mice were analyzed by qPCR at different time points following in vitro infection (Fig. 2a). Prior to infection, IL-1β mRNA was not detected in peritoneal Mϕ from either group of animals. Following infection, Mϕ derived from WT mice exhibited a higher mRNA level of IL-1β 48 and 72 h post infection, while the IL-1β mRNA level from the TLR2/9^−/−^ mice showed a slight increase with a peak at 48 h of infection; however, this level was lower than that in the WT mice and was followed by a decrease at the next time point. These results showed that the absence of TLR2 and TLR9 has a strong negative effect on IL-1β mRNA level. Thus, to identify the major IL-1β-producing cell in the TG and spleen of WT and TLR2/9^−/−^ mice, we performed flow cytometry analysis (Fig. 2b, c and Additional file 1: Figure S1). Our data showed that the main IL-1β response against infection occurs in the TG (Fig. 2b, left and Additional file 1: Figure S1a), as indicated by the high % of cells producing this cytokine in this tissue (with no significant differential detection in the spleen) (Fig. 2c and Additional file 1: Figure S1b) and that myeloid cells derived from TLR2/9^−/−^ mice were unable to produce this cytokine, as shown by the impaired production in the TG observed in mock-infected and infected mice (Fig. 2b, left). Since no detectable production of IL-1β by myeloid cells was seen in the TG of TLR2/9^−/−^ mice, we performed the subsequently analysis (Fig. 2b, right) only in the WT group. Thus, among the cell populations evaluated, it was discovered that DCs (Fig. 2b, right) are the major cell producing IL-1β in the TG during infection.

### Monocytes/macrophages are the most important producer of iNOS and have a pivotal role against HSV-1

To determine the primary tissue producing iNOS, which is essential for establishing an efficient immune response against HSV-1, a previous study from our group evaluated the levels of iNOS in the TG and brain of WT and TLR2/9^−/−^ mice through qPCR and an immunofluorescence assay. The data suggested that iNOS is expressed in a TLR-dependent manner and identified Mo/Mϕ as the primary iNOS-producing cells important for mouse survival [6]. Thus, to evaluate the production of iNOS at the local and systemic levels and identify other types of cells producing this molecule, we checked iNOS production in the TG and spleen of WT and TLR2/9^−/−^ mice through flow cytometry analysis (Fig. 3a, b and Additional file 2: Figure S2).

Our results showed an increase in the % of myeloid cells producing iNOS in the TG of infected WT mice; this was unlike in TLR2/9^−/−^ mice, which exhibited no statistically difference in the % of cells producing this molecule during infection (Fig. 3a, left). On the other hand, spleens from both WT and TLR2/9^−/−^ infected mice presented a reduction in the % of myeloid cells producing iNOS (Fig. 3a, right). In addition, a greater percentage of iNOS-producing Mo/Mϕ was found in the TG of WT mice during infection compared to TLR2/9^−/−^ infected mice (Fig. 3b and Additional file 2: Figure S2a). The spleens from WT and TLR2/9^−/−^ infected mice presented similar profile of Mo/Mϕ producing iNOS (Fig. 3b and Additional file 2: Figure S2b).

### Type I interferon expression in the TG is higher in infected than in mock-infected WT and TLR2/9^−/−^ animals 5 days after infection

The mRNA expression level of type I IFNs during HSV-1 infection was evaluated in the TG of WT and TLR2/9^−/−^ mice on the 5^th^ day after infection using qPCR. In the TG of both the WT and TLR2/9^−/−^ infected groups, the levels of IFN-β mRNA increased relative to their respective control groups (Fig. 4). Nevertheless, no difference was observed between WT and TLR2/9^−/−^ infected mice. As the virus was administered intranasally and type I IFNs are generally expressed earlier, we also decided to evaluate their expression in the first few hours of infection in the lungs.

### The expression of Type I interferons in the first 3 and 24 h after infection in the lung, in contrast, is higher only in the WT animals

**Fig. 5:**
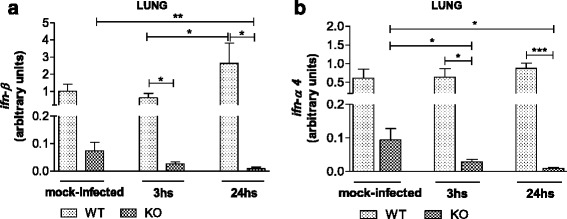
Earlier type I IFN expression in the lung occurs in WT infected animals. Mice were infected with 10^6^ p.f.u. of HSV-1, euthanized, and *lung tissues* were collected for mRNAs expression analysis by qPCR. IFN-β (**a**) and IFN-α4 (**b**) mRNAs level in the lung 3 or 24 h after infection. *Mock-infected* = animals that inhaled PBS. The results use arbitrary units for the ratio of target gene mRNA to endogenous control HPRT mRNA. Data are representative of two independent experiments with similar results. KO = TLR2/9^−/−^ mice. *Bars* show the means ± SEM. * *p* < 0.05; ** *p* < 0.01 and *** *p* < 0.001. *n* = 7–11 mice/group

The expression of type I IFNs mRNA in the lungs was measured 3 and 24 h after infection. The WT mice showed an increase in IFN-β mRNA level, which reached a higher level of expression at 24 h post infection, while the TLR2/9^−/−^ mice exhibited a marked decrease in the mRNA level of this cytokine at this time (Fig. 5a). A similar pattern in mRNA levels was also observed for IFN-α4 in the lungs of WT and TLR2/9^−/−^ infected mice (Fig. 5b).

### Infected mice generate more MCP-1 in the trigeminal ganglia and spleen than mock-infected mice

As reported previously, during HSV-1 infection, the chemokine MCP-1 is overexpressed in the TG of TLR2/9^−/−^ infected mice [8]. Thus, to better understand how the response of this protein occurs, MCP-1 expression and production in the TG, spleen and lung was evaluated.

**Fig. 6:**
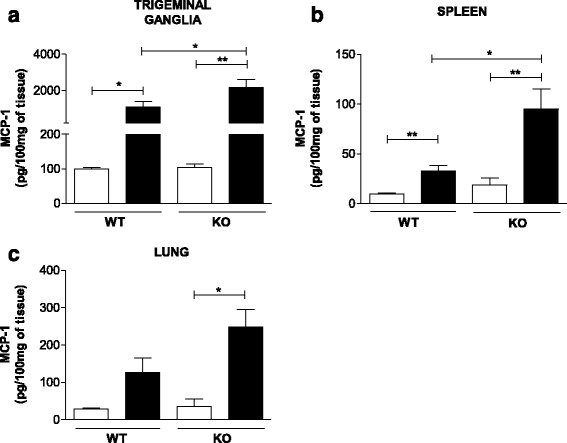
MCP-1 levels are higher in the TG and spleen of infected animals than mock-infected animals. C57BL/6 (WT) and TLR2/9^−/−^ (KO) mice were infected with 10^6^ p.f.u. of HSV-1, and the chemokine levels were determined in tissue homogenates with a bead-based immunoassay. **a** MCP-1 measured in the TG. **b** MCP-1 in the spleen. **c** MCP-1 in the lung. These experiments are representative of three independents experiments with similar results. *White bars*: mock-infected groups. *Black bars*: infected groups. *Bars* show means ± SEM. * *p* < 0.05 and ** *p* < 0.01

A higher level of MCP-1 was produced in the TG and spleen of WT infected mice relative to mock-infected mice. Higher production of MCP-1 was observed in the TLR2/9^−/−^ infected mice relative to WT infected mice (Fig. 6a). The same production profile was observed in the spleen (Fig. 6b). In the lung, the infected TLR2/9^−/−^ mice showed higher levels of MCP-1 than the mock-infected TLR2/9^−/−^ mice, whereas in WT mice, there was no differential production of this protein (Fig. 6c).

### Granzyme B is produced by CD8^+^ T and NK lymphocytes

**Fig. 7:**
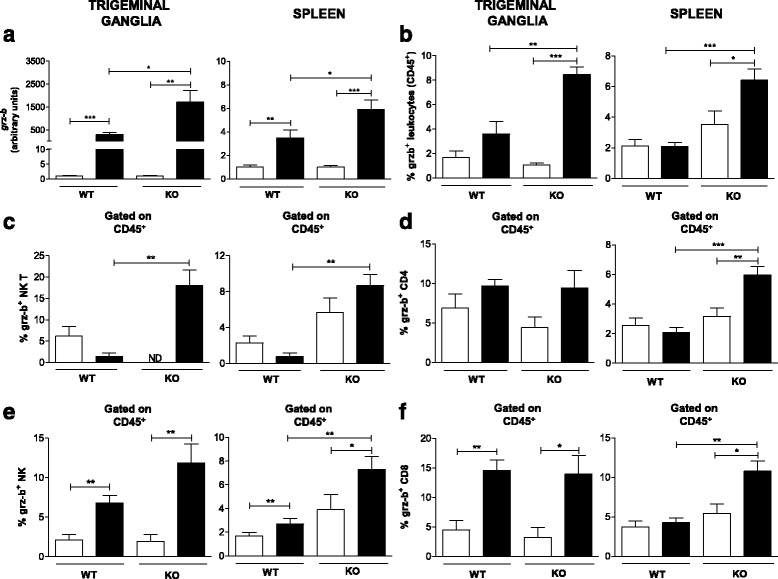
Granzyme B is produced in the TG of C57BL/6 mice by CD8^+^ T/NK after infection. **a** The GRZ-b mRNA level was measured in TG and spleen homogenates from C57BL/6 (WT) and TLR2/9^−/−^ (KO) mice on the 5^th^ day post infection (10^6^ p.f.u. of HSV-1) by qPCR. Data are representative of two independent experiments with similar results (*n* = 9–19 mice/group). The frequencies of GRZ-b-producing cells within the population of live leukocytes were analyzed by flow cytometry in the TG and the spleen **b**–**f** (6 animals per group). **b** GRZ-b^+^ NK T cells (CD45^+^CD3^+^NK1.1^+^); **c** GRZ-b^+^ CD4^+^ T cells (CD45^+^CD3^+^CD4^+^); **d** NK cells (CD45^+^NK1.1^+^); **e** GRZ-b^+^ CD8^+^ T cells (CD45^+^CD3^+^CD8^+^) and **f** GRZ-b^+^ live leucocytes (CD45^+^GRZb^+^). A minimum of 100,000 events was acquired for analysis. *White bars*: mock-infected groups. *Black bars*: infected groups. *ND*: not detected. *Bars* show the means ± SEM. * *p* < 0.05; ** *p* < 0.01 and *** *p* < 0.001

To better understand the mechanism underlying the immune response against HSV-1, the role of granzyme B during HSV-1 infection was also evaluated (Fig. 7). Therefore, we evaluated the mRNA levels of granzyme B in the TG and spleen of WT and TLR2/9^−/−^ mice and identified the cell types producing this molecule in these tissues during HSV-1 infection.

The mRNA level of granzyme B in the TG and spleen of infected mice was higher than that in mock-infected animals for both the WT and the TLR2/9^−/−^ animals, although the mRNA level in the infected TLR2/9^−/−^ mice was much higher than that in the WT mice (Fig. 7a). Thus, the next step was to identify the cells responsible for granzyme B production. Although the production of granzyme B in the spleen of WT infected compared to mock infected animals occurred distinctly only by NK cells, this molecule was also produced by other population of cells, though there was no statistical difference between the mock-infected and infected groups (Fig. 7c-f, right). The same was observed in the trigeminal ganglia (Fig. 7c-f, left). In this case, the production of granzyme B in WT animals occurred mainly through NK and CD8^+^ cells (Fig. 7e and f, left). However, in the TLR2/9^−/−^ mice, several cell types in the spleen, such as CD4^+^, CD8^+^, NK T and NK cells, produced granzyme B when we compared infected and mock-infected mice (Fig. 7c-f, right). Furthermore, analysis of a gate containing all granzyme B-positive cells confirmed that TG cells from TLR2/9^−/−^ mice not only show higher mRNA levels but also produce more granzyme B than WT mice, as observed for the spleen (Fig. 7b).

### CD8^+^ T lymphocytes are the main cell producing perforin in the spleen, while the perforin produced in the TG seems to be irrelevant

**Fig. 8:**
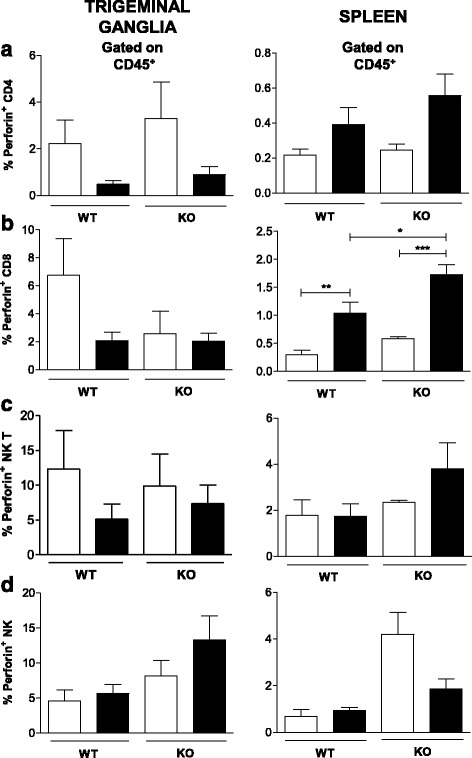
Perforin is produced by CD8^+^ T lymphocytes in the spleen of C57BL/6 mice after infection. Groups of C57BL/6 (WT) and TLR2/9^−/−^ (KO) mice (6 animals/group) were infected with 10^6^ p.f.u. HSV-1 via the intranasal route and, on the 5^th^ day post infection, mice were euthanized, and TG and spleen cell suspensions prepared for flow cytometry analysis. The frequency of each cell within the live leukocyte population was determined. **a** Frequencies of perforin-producing CD4^+^ T cells (CD45^+^CD3^+^CD4^+^); **b** CD8^+^ T cells (CD45^+^CD3^+^CD8^+^); **c** NK T cells (CD45^+^CD3^+^NK1.1^+^) and **d** NK cells (CD45^+^NK1.1^+^). A minimum of 100,000 events was acquired for analysis. *White bars*: mock-infected mice; *black bars*: infected mice. *Bars* show the means ± SEM. * *p* < 0.05; ** *p* < 0.01 and *** *p* < 0.001

Furthermore, the production of perforin was evaluated after HSV-1 infection. A comparison of the infected and mock-infected WT and TLR2/9^−/−^ groups showed that the overall production of perforin in the TG by several cell types (such as CD4^+^, CD8^+^, NK T and NK cells) was not statistically significant during infection (Fig. 8a-d). However, in the spleen, CD8^+^ T lymphocytes from the infected mice produced more perforin than the mock-infected cells. Additionally, the infected TLR2/9^−/−^ mice produced more perforin than the infected wild-type mice (Fig. 8b, right).

### TLR2/9^−/−^ mice exhibit a mix of Th1 and Th2 immune responses

Therefore, realizing that TLR2/9^−/−^ mice were less effective in protecting against HSV-1 [8] and that they produced less IL-1β and iNOS, typical molecules in the Th1 immune response, we analyzed the mRNA levels of IL-10, a regulatory cytokine in the immune response, and IL-5, an important component of the Th2 immune response, on the 5^th^ day post infection using qPCR. The infected WT mice exhibited a lower level of IL-5 mRNA and a higher level of IL-10 mRNA than the mock-infected WT mice but a much lower mRNA level of IL-10 than the infected TLR2/9^−/−^ mice.

**Fig. 9:**
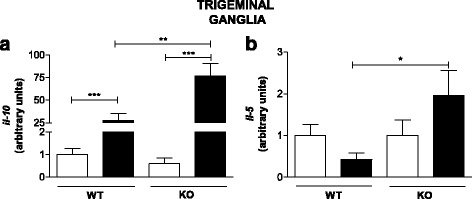
The immune response in TLR2/9^−/−^ mice appears to be a mix of Th1/ Th2 response. IL-10 **a** and IL-5 **b** mRNAs levels were measured in TG homogenates from C57BL/6 (WT) and TLR2/9^−/−^ (KO) mice on day 5 post infection (10^6^ p.f.u. of HSV-1) by qPCR. The expression results use arbitrary units for the ratio of target gene mRNA to the endogenous control, HPRT. Data are representative of two independent experiments with similar results. *n* = 9–19 mice/group. *White bars*: mock-infected groups. *Black bars*: infected groups. *Bars* show the means ± SEM. * *p* < 0.05; ** *p* < 0.01 and *** *p* < 0.001

Surprisingly, the TLR2/9^−/−^ infected mice exhibited an exacerbated response with regard to these two cytokines, indicating that a strong regulatory response was possibly being mediated by IL-10 due to the increased mRNA levels of chemokines in the TLR2/9^−/−^ mice (Fig. 9a). Additionally, a Th2-skewed response also occurred in the TLR2/9^−/−^ mice, which exhibited higher levels of IL-5 mRNA (Fig. 9b), suggesting a mixed Th1 and Th2 immune response in the TLR2/9^−/−^ mice.

## Discussion

Several studies have attempted to understand the main mechanisms involved in the immune response and identify the key components responsible for the recognition and establishment of an efficient immune response against HSV-1 [9, 15, 23–26]. Data from our group and from other studies have highlighted the key role of TLR2 and TLR9 in the recognition and activation of the immune response against HSV-1 infection and have suggested synergistic action of these receptors in the establishment of an effective response, since all the WT animals survive the infection, while all the TLR2/9^−/−^ mice die after infection [6, 8, 27, 28]. Furthermore, our group discovered that this immune response is mainly localized in the TG with the important role of the MyD88 adapter molecule along with IFN-γ and iNOS during infection. In the TG, after infection, a viral transcript and viral protein were identified [6, 8, 9]. Thus, continuing our studies, this work attempted to extend our knowledge of the immune response against HSV-1 by identifying the immune cells involved and the molecules produced as a result of their activation to obtain an effective response.

We showed that the % of Mo/Mϕ and DCs are higher in the TG of infected than mock-infected WT mice (Fig. 1b), which differs from what occurs in TLR2/9^−/−^ mice. These leukocytes are important components of the innate immune system as they play a crucial role in activating the antiviral cell-mediated immune response and also in producing important immune mediators [29–31]. During HSV-1 primary infection they act limiting viral replication and maintaining the latency state but also, activating the adaptive immune response [32]. Macrophages are especially important in phagocyte infected and apoptotic cells but also through the release of pro-inflammatory cytokines, as well as other immune mediators such as nitric oxide [33, 34]. Dendritic cells, in turn, are crucial for antigen presentation and, thus, trigger the adaptive immune response as was previously reported [35–38].

Furthermore, we showed that both the IL-1β mRNA [8] and protein level (Fig. 2b) are higher in the TG of infected than mock-infected WT mice. There was a drastic decrease in IL-1β in TLR2/9^−/−^ mice (Fig. 2b). This shows that IL-1β production is probably not down modulated by viral post-transcriptional modification, since HSV-1 is a complex virus with several evasion mechanisms, including inhibition of the translation of host proteins [39–41]. Importantly, it was discovered that among the studied cells, DCs in the TG of WT mice were the major cells producing IL-1β (Fig. 2b and Additional file 1: Figure S1a). As IL-1β is a cytokine with a major role to initiate the inflammatory process after an infection [42–44], this was also showed during the HSV-1 infection. Likewise, previous studies revealed that IL-1β deficiency is lethal to mice and demonstrated its important role in repairing pathological insults due to HSV-1 infection within the central nervous system [45, 46]. In TLR2/9^−/−^ mice the decreased IL-1β level could be one of the reasons for their highly mortality [8].

Zolini and collaborators (2014) [6], in addition to highlighting the TLR-dependence, showed that macrophages are the producers of iNOS, an important host defense protein [47, 48] in the TG. We confirmed that Mo/Mϕ were the primary iNOS-producing cells (Fig. 3a, b and Additional file 2: Figure S2) in the TG and that DCs were not good producers of iNOS. Other studies have also highlighted the important role of this molecule in host defense and indicate Mϕ as the main source, which is in line with our results [33, 34, 49, 50]. In other words, IL-1β together with iNOS is essential in mediating the local (TG), but not systemic (spleen), immune response against HSV-1. Additionally, these two molecules are TLR2/9 dependent, as TLR2/9^−/−^ mice produce them at low levels (Figs. 2 and 3), and, in contrast to WT mice, these mice died after infection [8].

IFN-β transcript, however, is detected in both WT and TLR2/9^−/−^ mice in the TG on the 5^th^ day after infection (Fig. 4), indicating that this response seems to be TLR-independent. However, at the beginning of the infection, the type I IFN (IFN-α and IFN-β) mRNA levels in the lung of WT mice were higher than those in the infected TLR2/9^−/−^ mice (Fig. 5a, b). As the response of these cytokines occurs early during a viral infection, their mRNA levels were down regulated in the lungs of TLR2/9^−/−^ mice in the first hours after intranasal infection. In other studies [32, 51–54] it was demonstrated that type I IFNs certainly play an essential role in the immune response in restricting viral infections and shaping the adaptive immune response.

Down regulation of the levels of IL-1β and iNOS in TG occurs simultaneously with overexpression of other molecules as MCP-1 and granzyme B in TLR2/9^−/−^ infected mice. The increase in MCP-1 mRNA levels after HSV-1 infection was already observed in the TG of TLR2/9^−/−^ mice in our previous study [8]; however, in the present work, the increase in MCP-1 was confirmed at the protein level in the TG and spleen as well (Fig. 6a, b). Thus, MCP-1 seems not to depend on TLRs to be expressed/produced. Alternatively, MCP-1 down regulation could depend on these receptors, since its mRNA and protein levels are higher in TLR2/9^−/−^ mice than WT mice after infection. As MCP-1 attracts monocytes, basophils, NK and T cells, its presence at these initial sites of HSV-1 infection could play a role in preventing the spread of virus to neighboring tissues [55], but these cells in TLR2/9^−/−^ mice lack functional TLR2 and TLR9, resulting in nonfunctional cells and in a nonfunctional immune response. Several studies have highlighted the relevance of MCP-1 in mediating the immune response against HSV-1, however its exactly role is not yet completely understood [25, 56–59].

Since our previous studies have indicated the importance of IFN-γ-producing CD8^+^ T cells in the control of HSV-1 infection in WT mice [8], we decided to evaluate the role of the granzyme B and perforin molecules and identify the primary cells producing these molecules. Notably, granzyme B (a serine protease) and perforin (a pore-forming protein) are important components of the immune response, as they confer the cytotoxic activity of NK cells and CD8^+^ T lymphocytes [60–62]. Moreover, these molecules, when released within the target cell, cause DNA fragmentation and rapid loss of membrane integrity, leading to cell apoptosis and, consequently, viral clearance [63, 64]. It was shown that granzyme B mRNA levels in the TG and spleen of infected WT mice were higher than those in mock-infected animals (Fig. 7a). The TLR2/9^−/−^ infected mice also showed a higher mRNA level of granzyme B than mock-infected mice and even a higher level than the WT mice. Thus, this high level of granzyme B in TLR2/9^−/−^ mice together with lower production of IL-1β and iNOS is not sufficient to control the infection, resulting in mortality (as reported in [8]). It was shown that among the studied cells, NK and CD8^+^ T cells are the major cells producing granzyme B in the TG, while in the spleen only the NK cells produced it (Fig. 7e, f). In TLR2/9^−/−^ mice TG large amount of granzyme B was produced by NK T, NK and CD8^+^ cells after infection (Fig. 7c, e-f). The production of granzyme B by NK T cells from the spleen of TLR2/9^−/−^ mice was even higher than that observed in the WT mice (Fig. 7c). In a more general analysis of the number of leukocytes producing granzyme B in the TG and spleen in the WT and TLR2/9^−/−^ infected mice, the production of granzyme B was higher in the TLR2/9^−/−^ mice than in the WT mice (Fig. 7b). Thus, in the WT infected mice, granzyme B could be mediating cell cytotoxic activity, regulating the immune response and interfering in HSV-1 reactivation in the TG, as previously evidenced in other studies [61, 65–67]. In contrast, in the infected TLR2/9^−/−^ mice, the high level of granzyme B could be contributing to an exacerbated immune response and the pathogenesis of the infection, contributing to animal death [68]. However, the regulation of perforin is different, with consistent basal production by all tested cells (CD4^+^, CD8^+^, NK T and NK cells) in the TG of mock-infected WT mice (Fig. 8a-d, left). This was also observed in the TG of TLR2/9^−/−^ mice (Fig. 8a-d, left). However, in the spleen, perforin was differentially produced by CD8^+^ T cells, which exhibited an increased % of perforin-positive cells after HSV-1 infection in both WT and TLR2/9^−/−^ mice (Fig. 8b, right). Thus, in the TG of WT mice, the basal levels of perforin seemed to be sufficient for its role in mediating cytotoxicity activity [63, 69, 70], or if the release of granzyme B occurs in a perforin-independent manner, it would not be necessarily functional [63, 71, 72]. However, in the TLR2/9^−/−^ mice, perforin possibly was not acting in viral clearance but contributing to the immunopathogenesis of infection, as previously described [63, 73, 74].

Another remarkable finding was that the IL-10 mRNA levels, which plays an important role in controlling the levels of pro-inflammatory cytokines [75–77] were higher in the TG of both WT and TLR2/9^−/−^ mice after HSV-1 infection (Fig. 9a). The mRNA levels were even higher in the TLR2/9^−/−^ mice than in the WT mice. Thus, IL-10 does not seem to depend on TLRs to increase its mRNA levels, or perhaps down regulation is TLR dependent. During HSV-1 infections, IL-10 was shown to control the production of immune mediators by microglial cells and corneal immunopathology in stromal keratitis [78–80]. Thus, the high levels of IL-10 mRNA observed in the TG of TLR2/9^−/−^ mice could be an attempt to control the exacerbated immune response triggered by the cells that were attracted by the high levels of MCP-1, whereas in the WT mice, its expression could be sufficient to control the immune response triggered by HSV-1. Surprisingly, increased levels of IL-5 mRNAs were observed in the TG of infected compared to mock-infected TLR2/9^−/−^ mice and also compared to WT infected mice (Fig. 9b). IL-5 is an important component of the Th2 immune response that is known to act not only in response to extracellular parasites and allergens but also in maintaining metabolic homeostasis and tissue repair [81, 82]. Type 2 immune responses are also known to play a regulatory role in limiting type 1 immune responses and the extent of its damage, as well [83]. IL-5, produced primarily by T helper type 2 cells and mast cells, induces B cell differentiation into antibody-producing cells and enhances the proliferation and differentiation of eosinophils [84, 85]. However, the production of IL-5 during an immune response can also lead to tissue damage, mainly through the excess activity of eosinophils [86, 87]. Therefore, the high IL-5 mRNA levels in the TG of TLR2/9^−/−^ infected mice could be contributing to their ineffective immune response.

## Conclusions

Altogether, this work has highlighted, among the studied cells, the main cells (DCs, Mo/Mϕ, CD8^+^ T lymphocytes) that produce the molecules (IL-1β; iNOS, granzyme B) in the key organ (TG) of the body that controls the immune response against HSV-1. Additionally, it was shown that, very early after infection, type I IFNs (IFN-α and IFN-β) are expressed by cells in virus-infected tissue and, also, that MCP-1 chemokine showed to be important in the HSV-1 response, since it attracts the immune cells to the site of infection. Beyond uncovering more knowledge about the host response to HSV-1, these data could help scientists develop efficient vaccines, treatments or diagnostic and prognostic approaches for HSV-1.

## Additional files

Additional file 1: Figure S1. Representative FACS density plots showing the gate strategy for the identification of IL-1β within CD11c^+^MHCII^high^ gated on live CD45^+^ leucocytes in the trigeminal ganglia (**a**) and spleen (**b**) from a single HSV1-infected WT mouse. A minimum of 100,000 events was acquired for analysis. (PPTX 386 kb)
Additional file 2: Figure S2. Representative FACS density plots showing the gate strategy for the identification of iNOS within F4/80^+^ gated on live CD45^+^ leucocytes in the trigeminal ganglia (**a**) and spleen (**b**) from a single HSV1-infected WT mouse. A minimum of 100,000 events was acquired for analysis. (PPTX 2700 kb)

## Abbreviations

CBA: Cytometric bead array
DCs: Dendritic cells
DMEM: Dulbecco’s Modified Eagle’s Medium
dpi: Day post infection
FACS buffer: PBS with 2% FBS
FBS: Fetal bovine serum
GRZ-b: Granzyme B
HPRT: Hypoxanthine-guanine phosphoribosyltransferase
HSV-1: Herpes simplex virus type 1
IFN: Interferon
IL: Interleukin
iNOS: Nitric oxide synthase
m.o.i.: Multiplicity of infection
MCP-1: Monocyte chemoattractant protein-1
Mo/Mϕ: Monocyte/macrophage
MyD88: Myeloid differentiation factor 88
Mϕ: Macrophage
NK: Natural killer
NO: Nitric oxide
p.f.u.: Plaque-forming unit
PAMPs: Pathogen-associated molecular patterns
PBS: Phosphate-buffered saline
PRRs: Pattern recognition receptors
qPCR: Quantitative PCR
RPMI: Roswell Park Memorial Institute medium
RT: Reverse transcription
TG: Trigeminal ganglia
TLR2/9^−/−^: TLR2 and TLR9 knockout
TLR2^−/−^: TLR2 knockout
TLR9^−/−^: TLR9 knockout
TLRs: Toll-like receptors
WT: Wild type
