# Supplementary material for: Dendritic cells, macrophages, NK and CD8+ T lymphocytes play pivotal roles in controlling HSV-1 in the trigeminal ganglia by producing IL1-beta, iNOS and granzyme B
Source: Virol J. 2017 Feb 21;14:37. doi: 10.1186/s12985-017-0692-x (PMC5320739; doi:10.1186/s12985-017-0692-x)
Supplement: Additional file 1: Figure S1. — Representative FACS density plots showing the gate strategy for the identification of IL-1β within CD11c+MHCIIhigh gated on live CD45+ leucocytes in the trigeminal ganglia (a) and spleen (b) from a single HSV1-infected WT mouse. A minimum of 100,000 events was acquired for analysis. (PPTX 386 kb) [file 12985_2017_692_MOESM1_ESM.pptx]

## Slide 1
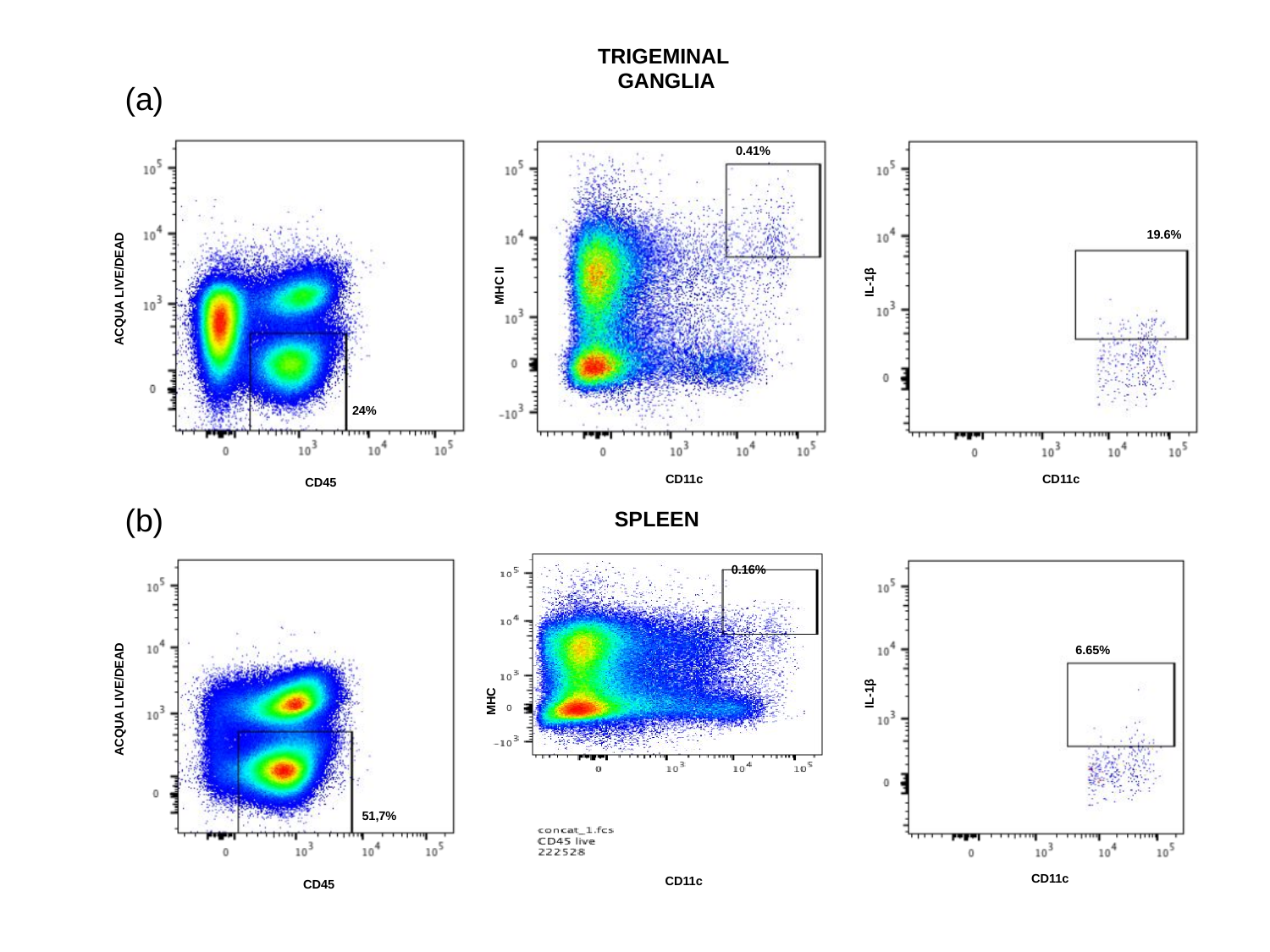

TRIGEMINAL
GANGLIA
(a)
0.41%
19.6%
IL-1β
MHC II
ACQUA LIVE/DEAD
24%
CD11c
CD11c
CD45
(b)
SPLEEN
0.16%
6.65%
IL-1β
ACQUA LIVE/DEAD
MHC
51,7%
CD11c
CD11c
CD45
