# Supplementary material for: Dendritic cells, macrophages, NK and CD8+ T lymphocytes play pivotal roles in controlling HSV-1 in the trigeminal ganglia by producing IL1-beta, iNOS and granzyme B
Source: Virol J. 2017 Feb 21;14:37. doi: 10.1186/s12985-017-0692-x (PMC5320739; doi:10.1186/s12985-017-0692-x)
Supplement: Additional file 2: Figure S2. — Representative FACS density plots showing the gate strategy for the identification of iNOS within F4/80+ gated on live CD45+ leucocytes in the trigeminal ganglia (a) and spleen (b) from a single HSV1-infected WT mouse. A minimum of 100,000 events was acquired for analysis. (PPTX 2700 kb) [file 12985_2017_692_MOESM2_ESM.pptx]

## Slide 1
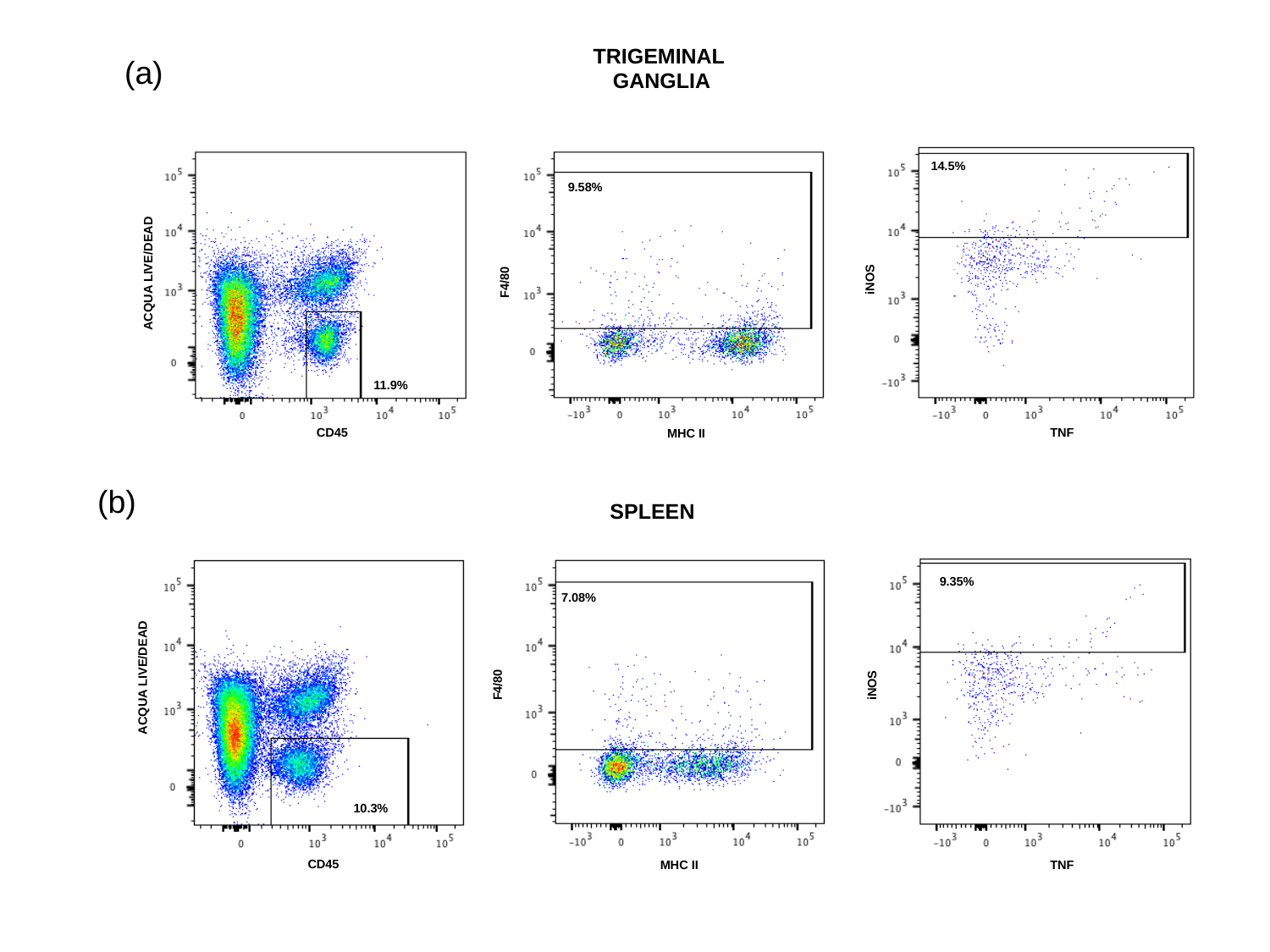

TRIGEMINAL
GANGLIA
(a)
14.5%
9.58%
ACQUA LIVE/DEAD
iNOS
F4/80
11.9%
TNF
CD45
MHC II
(b)
SPLEEN
9.35%
7.08%
ACQUA LIVE/DEAD
F4/80
iNOS
10.3%
CD45
MHC II
TNF
